# Correction: Machado et al. The Effect of Curcumin on Renal Ischemia/Reperfusion Injury in Diabetic Rats. *Nutrients* 2022, *14*, 2798

**DOI:** 10.3390/nu14224835

**Published:** 2022-11-15

**Authors:** Douglas Ikedo Machado, Eloiza de Oliveira Silva, Sara Ventura, Maria de Fátima Fernandes Vattimo

**Affiliations:** School of Nursing, University of São Paulo, São Paulo 05403-000, Brazil

In the original publication [1], the funder **Coordenação de Aperfeiçoamento de Pessoal de Nível Superior—Brasil (CAPES)**, **Finance Code 001** was not included. 

**Corrected Funding:**

This research was funded by São Paulo State Research Support Foundation FAPESP, Grant Number 2019/03415-3, and Coordenação de Aperfeiçoamento de Pessoal de Nível Superior—Brasil (CAPES), Finance Code 001.

The authors apologize for any inconvenience caused and state that the scientific conclusions are unaffected. This correction was approved by the Academic Editor. The original publication has also been updated.
